# Leishmania donovani Attenuates Dendritic Cell Trafficking to Lymph Nodes by Inhibiting C-Type Lectin Receptor 2 Expression via Transforming Growth Factor-β

**DOI:** 10.1128/spectrum.04122-22

**Published:** 2023-05-01

**Authors:** Manisha Yadav, Md. Naushad Akhtar, Manish Mishra, Sandeep Kumar, Raj Kumar, Shubham, Anil Nandal, Pradip Sen

**Affiliations:** a Division of Cell Biology and Immunology, Council of Scientific and Industrial Research—Institute of Microbial Technology, Chandigarh, India; b Academy of Scientific and Innovative Research (AcSIR), Ghaziabad, India; University of Arkansas for Medical Sciences

**Keywords:** dendritic cell trafficking, CLEC-2, *Leishmania donovani*, c-Src, TGF-β, NF-κB

## Abstract

To initiate an antileishmanial adaptive immune response, dendritic cells (DCs) must carry *Leishmania* antigens from peripheral tissues to local draining lymph nodes. However, the migratory capacity of DCs is largely compromised during Leishmania donovani infection. The molecular mechanism underlying this defective DC migration is not yet fully understood. Here, we demonstrate that L. donovani infection impaired the lymph node homing ability of DCs by decreasing C-type lectin receptor 2 (CLEC-2) expression. L. donovani exerted this inhibitory effect by inducing transforming growth factor-β (TGF-β) secretion from DCs. Indeed, TGF-β produced in this manner inhibited nuclear factor-κB (NF-κB)-mediated CLEC-2 expression on DCs by activating c-Src. Notably, suppression of c-Src expression significantly improved the arrival of DCs in draining lymph nodes by preventing L. donovani-induced CLEC-2 downregulation on DCs. These findings reveal a unique mechanism by which L. donovani inhibits DC migration to lymph nodes and suggest a key role for TGF-β, c-Src, and CLEC-2 in regulating this process.

**IMPORTANCE** Dendritic cells (DCs) play a key role in initiating T cell-mediated protective immunity against visceral leishmaniasis (VL), the second most lethal parasitic disease in the world. However, the T cell-inducing ability of DCs critically depends on the extent of DC migration to regional lymph nodes. Notably, the migration of DCs is reported to be impaired during VL. The cause of this impaired DC migration, however, remains ill-defined. Here, we provide the first evidence that L. donovani, the causative agent of VL, attenuates the lymph node homing capacity of DCs by decreasing C-type lectin receptor 2 (CLEC-2) expression on DCs. Additionally, we have demonstrated how L. donovani mediates this inhibitory effect. Overall, our work has revealed a unique mechanism underlying L. donovani-induced impairment of DC migration and suggests a potential strategy to improve antileishmanial T cell activity by increasing DC arrival in lymph nodes.

## INTRODUCTION

Visceral leishmaniasis (VL), caused by Leishmania donovani, is regarded as the world’s second-deadliest parasitic disease after malaria (1). The protective immune response largely relies on the activity of dendritic cells (DCs), which play a crucial role in initiating antileishmanial T cell responses (2). However, to initiate antileishmanial T cell reactivity, DCs must ferry *Leishmania* antigens from peripheral tissues to the T cell areas of draining lymph nodes (3). Notably, DC migration is reported to be impaired during chronic L. donovani infection, which keeps DCs and T cells spatially apart and thereby promotes immunosuppression and disease development (4). One proposed explanation for this defective DC migration is that the expression of CCR7 (a chemokine receptor) on DCs decreases during L. donovani infection (4). As a result, the responsiveness and migratory capacity of DCs to CCL21 and CCL19 (CCR7 ligands) are significantly impaired in chronically infected mice (4). However, since DC migration is also mediated by other receptors (5), the possibility of an alternate receptor being involved in impaired DC migration during L. donovani infection cannot be ruled out. Accordingly, how L. donovani regulates DC migration remains a question of central interest.

Previously, C-type lectin receptor-2 (CLEC-2) was identified as a key receptor required for DC homing to lymph nodes. Indeed, mice lacking CLEC-2 display impaired DC trafficking to lymph nodes and attenuated T cell response (6). CLEC-2 supports the migration of DCs to draining lymph nodes by interacting with its ligand podoplanin (PDPN), which is expressed throughout the lymphatic route (6). Although CLEC-2 serves as an important receptor for DC trafficking, its role in regulating DC migration during L. donovani infection remains unaddressed. Recently, we demonstrated that the anti-inflammatory cytokine transforming growth factor-β (TGF-β) inhibits CLEC-2-mediated DC migration (5). Furthermore, TGF-β is reported to be expressed during L. donovani infection (7). Accordingly, in the current study, we investigated the role of CLEC-2 in L. donovani-induced inhibition of DC migration. Specifically, we examined the following: whether L. donovani regulates CLEC-2 expression on DCs and, if so, whether TGF-β has any role in this process; how L. donovani regulates CLEC-2 expression on DCs; and whether CLEC-2 contributes to defective DC migration during L. donovani infection. Finally, we determined the relevance of the molecular pathway via which L. donovani regulates CLEC-2 expression on DCs in L. donovani-induced impairment of DC migration to the lymph nodes. Here, we show that L. donovani inhibits DC migration to the lymph nodes by reducing CLEC-2 expression on DCs and that TGF-β plays an important role in mediating this immunoregulatory effect.

## RESULTS

### L. donovani downregulates CLEC-2 expression on DCs in a TGF-β-dependent manner.

**FIG 1:**
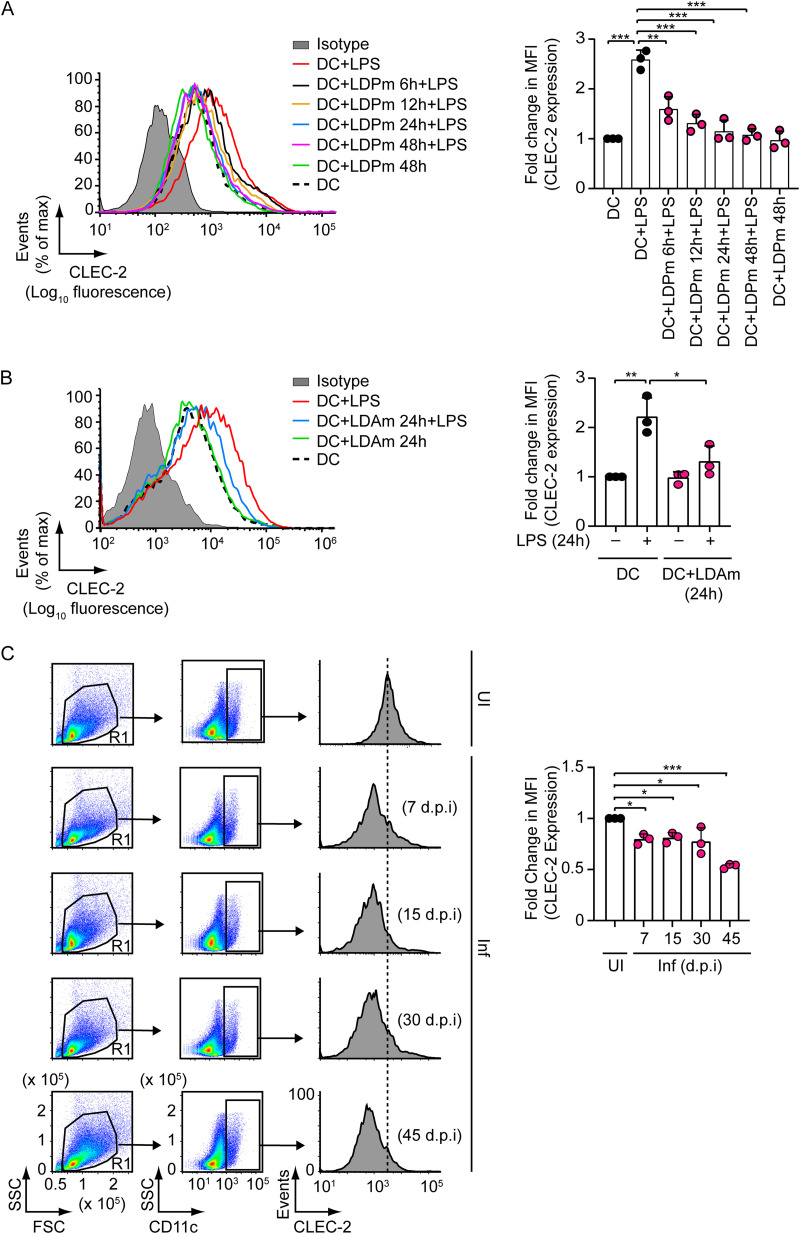
L. donovani inhibits LPS-stimulated CLEC-2 expression on DCs. (A and B) BMDCs were infected with L. donovani promastigotes (LDPm; A) or amastigotes (LDAm; B) for specified times and then stimulated (for 24 h) with LPS. CLEC-2 expression on BMDCs was assessed via flow cytometry. Data are representative of those from three independent experiments (left panel). (Right panel) Relative mean fluorescence intensity (MFI) data of CLEC-2 expression pooled from three separate experiments. The mean fluorescence intensity of CLEC-2 expression was measured after subtracting the background signals (isotype control) and presented here as fold change relative to results for uninfected DCs left unstimulated. (C) Expression of CLEC-2 on sDCs (i.e., CD11c-gated spleen cells) from uninfected (UI) mice or L. donovani-infected (Inf) mice, analyzed via flow cytometry on indicated days postinfection (d.p.i). Data are representative of those from three independent experiments (left panel). FSC, forward scatter; SSC, side scatter. (Right panel) Relative mean fluorescence intensity of CLEC-2 expression (calculated as for panel A) compiled from three experiments. In all panels, error bars represent SD, and each symbol in the graphs corresponds to data derived from an individual experiment. ***, *P < *0.001; **, *P < *0.01; *, *P < *0.05.

To determine the effect of L. donovani on CLEC-2 expression by DCs, we infected bone marrow-derived DCs (BMDCs) with L. donovani promastigotes (extracellular form; LDPm) for various times (6 h to 48 h), treated them with lipopolysaccharide (LPS) for 24 h, and analyzed CLEC-2 expression by flow cytometry. Upon LPS stimulation, CLEC-2 expression was substantially upregulated on BMDCs (Fig. 1A). LDPm infection, on the other hand, caused a temporal decrease in LPS-stimulated CLEC-2 expression on BMDCs, with maximum inhibition observed at 24 h postinfection and lasting up to 48 h (Fig. 1A; see also Fig. S1 in the supplemental material for DC infection status). Next, we checked whether L. donovani amastigotes (intracellular form; LDAm) exhibited a similar inhibitory effect on CLEC-2 expression by BMDCs. For this, we infected BMDCs with LDAm for 24 h, as this was the initial time point of LDPm infection at which we observed the largest reduction in LPS-stimulated CLEC-2 expression (Fig. 1A). We then stimulated BMDCs with LPS for 24 h and assessed CLEC-2 expression on BMDCs via flow cytometry. As expected, we observed a marked decrease in LPS-stimulated CLEC-2 expression on BMDCs after LDAm infection (Fig. 1B). Thus, both extracellular and intracellular forms of L. donovani suppress LPS-induced CLEC-2 upregulation on DCs.

We further conducted a kinetic study to examine if and when CLEC-2 expression is reduced on splenic DCs (sDCs; defined by CD11c^+^ cells [8–13]) in L. donovani-infected mice. We found that CLEC-2 expression on sDCs began to decline within 7 days postinfection and continued to decrease gradually for at least 45 days (Fig. 1C). Notably, the time points 7, 15, and 30 days postinfection correspond to the three stages of infection, namely, the first appearance of amastigotes in the spleen, the establishment of infection, and the chronic phase of infection, respectively (14, 15). Thus, our results suggest that DCs derived from the spleen (a visceral organ) of L. donovani-infected mice exhibit lower CLEC-2 expression from the early phase of infection and that this lower expression of CLEC-2, along with CCR-7 (4), may contribute to impaired DC migration to splenic lymphoid tissue. Then, we went on to examine the expression of PDPN (CLEC-2 ligand) in L. donovani-infected mice. In contrast to uninfected mice, L. donovani-infected mice displayed a progressive increase in the PDPN-expressing lymphatic endothelial cell (LEC) frequency from day 15 to at least day 45 postinfection (Fig. S2).

**FIG 2:**
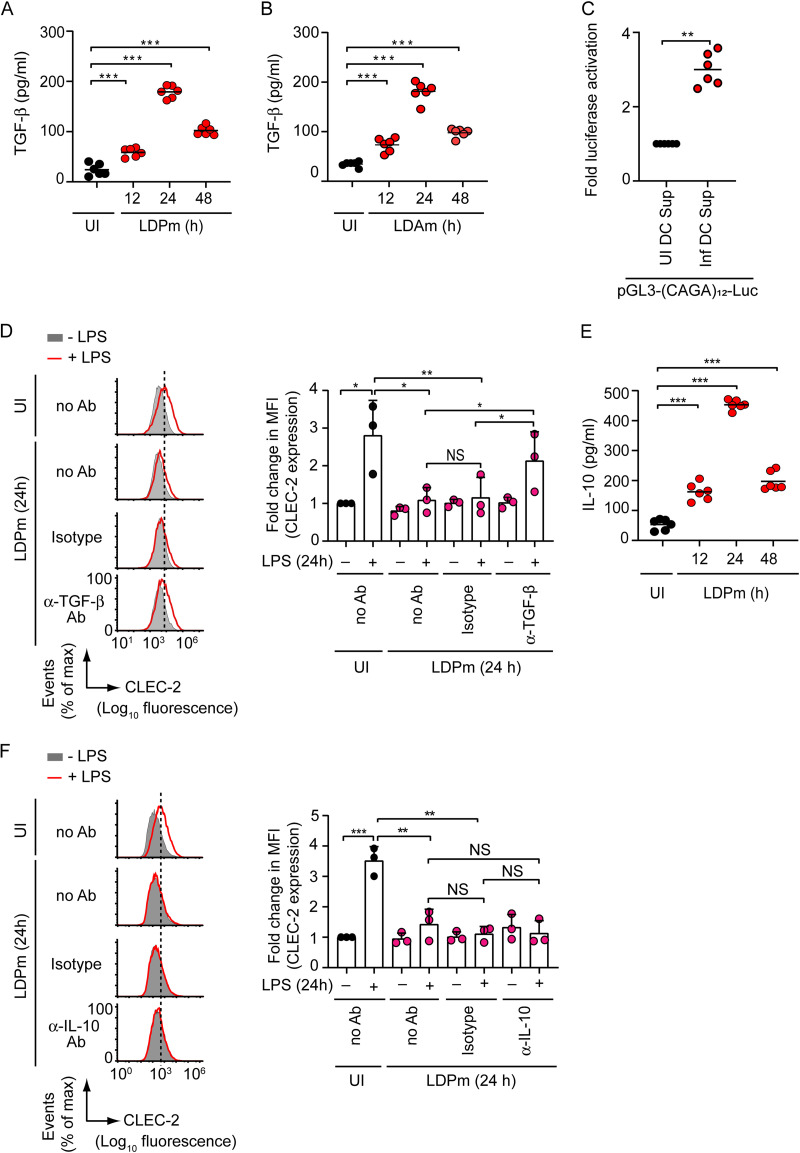
L. donovani-induced TGF-β production inhibits LPS-stimulated CLEC-2 expression on DCs. (A and B) BMDCs were infected with LDPm (A) or LDAm (B) for the indicated times, and TGF-β secretion was measured via ELISA. Data are a compilation of those from two separate experiments (*n *= 3 in each experiment). (C) Bioactivity assay of TGF-β produced by LDPm-infected BMDCs. HEK-293T cells were transfected with a SMAD-responsive luciferase reporter construct [pGL3-(CAGA)_12_-Luc] plus *Renilla* luciferase plasmid (pRL-CMV; internal control) and then incubated for 24 h with supernatants derived from uninfected BMDC culture (UI DC Sup) or 24-h LDPm-infected BMDC culture (Inf DC Sup). The luciferase activity was measured with a dual-luciferase reporter assay system. Results were normalized to *Renilla* luciferase activity and are presented relative to those for the cells transfected with pGL3-(CAGA)_12_-luciferase reporter construct and incubated with uninfected BMDC culture supernatant. Data are a compilation of those from three separate experiments (*n *= 2 in each experiment). (D) BMDCs were infected with LDPm in the presence of no antibody (no Ab), isotype control Ab, or anti-TGF-β Ab for 24 h and then treated with LPS for an additional 24 h. The expression of CLEC-2 on BMDCs was analyzed via flow cytometry. Data are representative of those from three independent experiments (left panel). (Right panel) Combined data (from three different experiments) of the relative mean fluorescence intensity of CLEC-2 expression. The mean fluorescence intensity of CLEC-2 expression was calculated as for Fig. 1A. (E) BMDCs were infected with LDPm as for panel A, and IL-10 secretion was measured via ELISA. Data are a compilation of two separate experiments (*n *= 3 in each experiment). (F) BMDCs were infected with LDPm in the absence (no Ab) or presence of isotype control Ab or anti-IL-10 Ab for 24 h and stimulated with LPS for 24 h. The CLEC-2 level on BMDCs was evaluated via flow cytometry. Data are representative of those from three independent experiments (left panel). (Right panel) Relative mean fluorescence intensity values of CLEC-2 expression (calculated as for Fig. 1A) pooled from three different analyses are presented graphically. Error bars indicate SD. Each symbol represents data of an individual replicate (A to C and E) or one independent experiment (D and F), and the bars indicate means. ***, *P < *0.001; **, *P < *0.01; *, *P < *0.05. NS, not significant.

After this, we investigated whether TGF-β contributes to L. donovani-induced inhibition of CLEC-2 expression on DCs. We explored this possibility because our previous study showed that TGF-β inhibits CLEC-2 expression on DCs (5). Furthermore, TGF-β expression is known to be upregulated during L. donovani infection (7). Initially, we determined whether L. donovani induces TGF-β secretion from DCs. Our results demonstrated that LDPm infection increased TGF-β secretion from BMDCs, which peaked at 24 h after infection and decreased after 48 h (Fig. 2A). We noted enhanced TGF-β production by BMDCs also after LDAm infection (Fig. 2B). We then tested whether this produced TGF-β was biologically active. For these experiments, we transfected HEK-293T cells with a luciferase reporter plasmid containing 12 repeats of the TGF-β-responsive SMAD-binding element (CAGA) (16–19). Subsequently, we cultured HEK-293T cells for 24 h in the presence of supernatants derived from uninfected or LDPm-infected BMDC culture and measured luciferase activity using the dual-luciferase reporter assay kit. Compared to uninfected BMDC culture supernatants, LDPm-infected BMDC culture supernatants stimulated more luciferase activity in HEK-293T cells (Fig. 2C). This finding validated the biological activity of TGF-β produced by L. donovani-infected BMDCs. Next, to verify the role of TGF-β in L. donovani-induced inhibition of CLEC-2 expression on DCs, we analyzed the effect of TGF-β neutralization with anti-TGF-β antibody (Ab). Unlike isotype control Ab, anti-TGF-β Ab significantly reduced the inhibitory effect of LDPm on LPS-stimulated CLEC-2 expression on BMDCs (Fig. 2D). Thus, TGF-β plays an essential role in L. donovani-induced inhibition of CLEC-2 expression on DCs. We further observed that LDPm infection stimulated the secretion of another anti-inflammatory cytokine, interleukin-10 (IL-10), from BMDCs (Fig. 2E). In addition, we have recently demonstrated an increased IL-10 production by BMDCs following LDAm infection (20). Accordingly, we verified whether IL-10 also plays a role in L. donovani-mediated suppression of CLEC-2 expression on DCs. Interestingly, we found no effect of IL-10 neutralization with anti-IL-10 Ab on LDPm-induced inhibition of CLEC-2 expression on DCs (Fig. 2F). This is consistent with our previous finding that IL-10 does not influence CLEC-2 expression on DCs (5). Collectively, our results demonstrate that L. donovani inhibits CLEC-2 expression on DCs through TGF-β.

### L. donovani-induced CLEC-2 downregulation via TGF-β inhibits DC migration.

**FIG 3:**
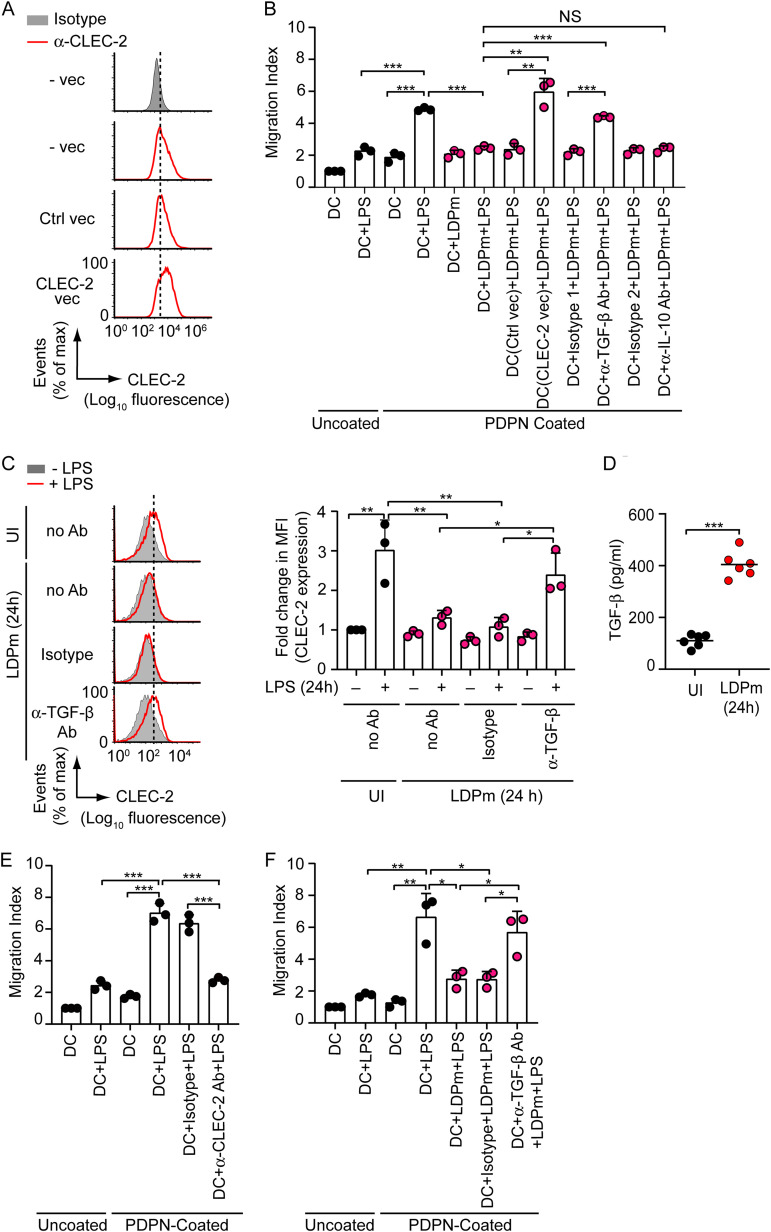
Roles of CLEC-2 and TGF-β in L. donovani-mediated regulation of DC migration toward PDPN. (A) Flow cytometry analysis of CLEC-2 expression on BMDCs left untransfected (vec) or transfected with control vector (Ctrl vec) or CLEC-2-expressing vector (CLEC-2 vec). Data are representative of those from two different experiments. (B) BMDCs were infected with LDPm for 24 h in the presence or absence of neutralizing anti-TGF-β or anti-IL-10 Ab or respective isotype control Abs (isotype-1, mouse IgG1,κ [isotype control Ab for anti-TGF-β]; isotype-2, rat IgG2b,κ [isotype control Ab for anti-IL-10]). Alternatively, BMDCs were transfected with plasmid vectors as for panel A before LDPm infection. BMDCs were then treated with LPS for an additional 24 h. The migration of BMDCs toward PDPN was analyzed using a Transwell system whose lower well was coated with PDPN. Uncoated, uncoated wells; PDPN-coated, PDPN-coated wells. Data are a compilation of those from three independent experiments. (C) Flow cytometry analysis of CLEC-2 expression on HuMoDCs infected with LDPm for 24 h in the presence or absence (no Ab) of isotype control Ab or anti-TGF-β Ab and then treated for 24 h with LPS. Data are representative of those from three independent experiments (left panel). (Right panel) Combined data from three experiments for relative mean fluorescence intensity of CLEC-2 expression calculated as for Fig. 1A. (D) ELISA of TGF-β production by HuMoDCs infected with LDPm for 24 h. Data from two independent experiments (*n* = 3 per experiment) are presented. (E and F) HuMoDCs were treated with LPS for 24 h in the presence or absence of isotype control Ab or anti-CLEC-2 Ab or left untreated (E). Alternatively, HuMoDCs were infected (for 24 h) with LDPm in the presence or absence of isotype control Ab or anti-TGF-β Ab and then cultured with LPS for 24 h (F). The migration of HuMoDCs toward the PDPN-coated wells was assessed using a Transwell system as described for panel B. Data are a compilation of those from three separate experiments. Error bars indicate SD. Each symbol represents data from an independent experiment (B, C [right panel], E, and F) or a single replicate (D), and the bars indicate means. ***, *P < *0.001; **, *P < *0.01; *, *P < *0.05.

As stated above, efficient DC migration requires the binding of CLEC-2 to its ligand PDPN, which is expressed on the lymphatic surface (6). This information prompted us to investigate whether L. donovani-induced inhibition of CLEC-2 expression affects the migratory ability of DCs toward PDPN. In addition, we examined the role of TGF-β in mediating this process. Accordingly, we performed a Transwell migration assay using a 24-well Transwell plate in which the lower wells were coated with PDPN-Fc protein. As shown in Fig. 3A and B, LPS treatment increased the migratory ability of BMDCs toward PDPN-coated wells. However, infection with LDPm inhibited this effect. Overexpression of CLEC-2 in BMDCs or neutralization of TGF-β with anti-TGF-β Ab, on the other hand, considerably improved the migratory ability of LPS-treated BMDCs despite LDPm infection. Unlike TGF-β neutralization, IL-10 neutralization (with anti-IL-10 Ab) did not reverse the inhibitory effect of L. donovani on the migration of LPS-treated BMDCs. These results suggest that L. donovani reduces BMDC migration toward PDPN by downregulating CLEC-2 expression on DCs and that TGF-β, but not IL-10, produced by DCs during L. donovani infection plays a pivotal role in mediating this inhibitory effect.

Then, we determined whether L. donovani similarly reduces CLEC-2 expression on human monocyte-derived DCs (HuMoDCs) in a TGF-β-dependent manner, thereby inhibiting HuMoDC migration toward PDPN. Our results showed that similar to the case with murine DCs, LDPm inhibited LPS-stimulated CLEC-2 upregulation on HuMoDCs (Fig. 3C). However, treatment with anti-TGF-β Ab reduced this inhibitory effect of LDPm (Fig. 3C). These results indicate that TGF-β is required for L. donovani-induced inhibition of CLEC-2 expression on HuMoDCs. Indeed, we observed increased TGF-β production in HuMoDCs after LDPm infection (Fig. 3D). Our results from the Transwell migration assay further showed that LPS stimulation enhanced HuMoDC migration toward PDPN-coated wells (Fig. 3E). Treatment of HuMoDCs with anti-CLEC-2 Ab, however, prevented this effect (Fig. 3E). Notably, the migration of LPS-treated HuMoDCs to PDPN-coated wells was also inhibited by LDPm infection (Fig. 3F). In contrast, neutralization of TGF-β with anti-TGF-β Ab restored the increased migration of LPS-treated HuMoDCs despite LDPm infection (Fig. 3F). Thus, our findings based on murine and human DCs suggest that L. donovani inhibits DC migration by reducing CLEC-2 expression via TGF-β.

### L. donovani suppresses CLEC-2 upregulation on DCs by inhibiting NF-κB through TGF-β.

**FIG 4:**
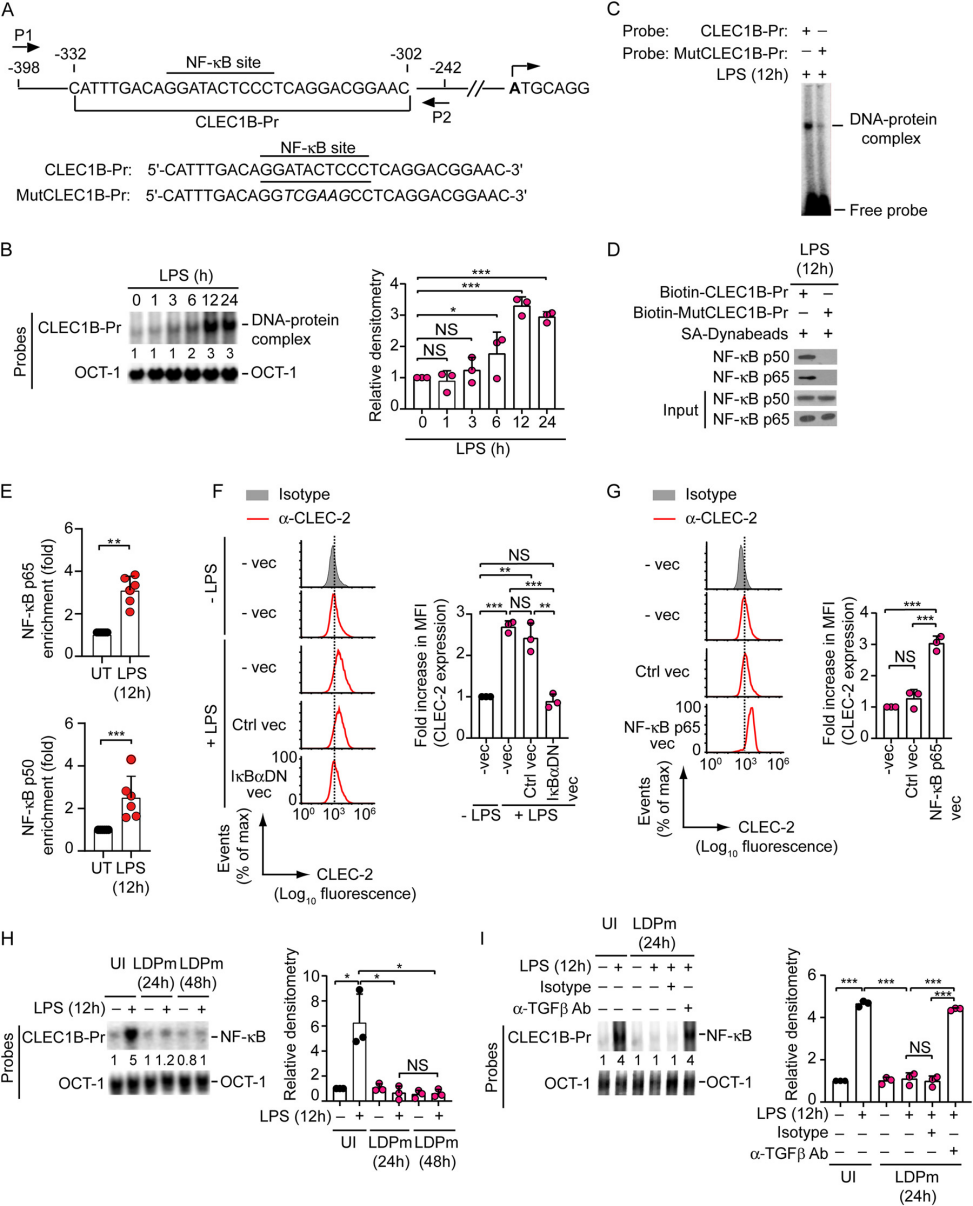
L. donovani-induced TGF-β production suppresses NF-κB-driven CLEC2 upregulation on DCs. (A) Schematic of mouse *CLEC1B* promoter showing the positions of putative NF-κB-binding site and the primers (P1 and P2) used for ChIP analysis, and details of oligonucleotide probes used for EMSA. Mouse *CLEC1B* promoter-specific CLEC1B-Pr probe contains putative wild-type NF-κB-binding sites, and MutCLEC1B-Pr probe contains mutations (italicized) at NF-κB-binding site. Base positions are relative to the ATG (translation) start site. (B) EMSA of nuclear extracts of BMDCs treated with LPS for specified times and analyzed with indicated probes. Numbers below lanes indicate densitometry (normalized to OCT-1 binding [internal control]) relative to that of untreated DCs (0 h). Data are representative of three independent experiments (left panel). (Right panel) Relative densitometry results from three separate experiments. (C) Nuclear extracts from BMDCs treated with LPS for 12 h were subjected to EMSA using CLEC1B-Pr and MutCLEC1B-Pr probes, which contained wild-type and mutated NF-κB sites, respectively. Data are representative of those from three independent experiments. (D) Pulldown assay was performed for nuclear proteins derived from LPS-treated (for 12 h) BMDCs using streptavidin (SA)-conjugated Dynabeads and indicated biotin-labeled oligonucleotides. The bound proteins were immunoblotted with antibodies against p65 and p50 subunits of NF-κB. Input represents nuclear extracts (5%) before pulldown. Data are representative of those from three independent experiments. (E) Recruitment of p65 and p50 subunits of NF-κB to the mouse *CLEC1B* promoter was analyzed (via ChIP-quantitative real-time PCR analysis; primer details are given in Table S1, and the location of primers is indicated in panel A) in BMDCs after treatment with LPS for 12 h. Results are a compilation of those from three experiments (*n *= 2 per experiment), presented as fold enrichment relative to that of untreated (UT) BMDCs. (F) BMDCs were transfected with control vector or IκBα dominant negative (IκBαDN) vector or left untransfected (-vec). BMDCs were then treated with LPS for 24 h. The expression of CLEC-2 on BMDCs was analyzed via flow cytometry. Data are representative of three separate analyses (left panel). Right panel: bar graph shows the relative mean fluorescence intensity of CLEC-2 expression (measured as Fig. 1A) from three separate analyses. (G) BMDCs were transfected or not (-vec) with control or NF-κB p65-encoding vector. Flow cytometry analysis was performed to assess the surface expression of CLEC-2 on BMDCs. Data are representative of three individual experiments (left panel). (Right panel) Combined data of relative mean fluorescence intensity of CLEC-2 expression (calculated as for Fig. 1A) from three experiments. (H) BMDCs were infected with LDPm for 24 or 48 h or kept uninfected (UI) and then treated (+) or not (−) with LPS for 12 h. EMSA was performed with the indicated probes. Numbers below lanes indicate densitometry of NF-κB binding (normalized to OCT-1 binding). Data are representative of those from three independent experiments (left panel). (Right panel) Relative densitometry data (pooled from three different experiments). (I) Binding of NF-κB to *CLEC1B* promoter in BMDCs left uninfected or infected with LDPm for 24 h in the absence (−) or presence (+) of isotype control Ab or anti-TGF-β Ab, and then treated (or not) with LPS for 12 h, analyzed via EMSA using indicated probes. Numbers below lanes indicate densitometry (as in panel H), presented relative to uninfected BMDCs that were cultured in the absence of Ab and given no LPS treatment. Data are representative of those from three independent experiments (left panel). (Right panel) Combined densitometry data (relative) from three different experiments. Error bars indicate SD. Each symbol in the graphs corresponds to data derived from one independent experiment (right panels of B, F, G, H, and I) or an individual replicate (E). ***, *P < *0.001; **, *P < *0.01; *, *P < *0.05.

We then directed our effort to determine how L. donovani-induced TGF-β suppressed LPS-stimulated CLEC-2 expression on DCs. In this context, we initially sought to identify the mechanism that promoted LPS-induced CLEC-2 upregulation on BMDCs. Since nuclear factor-κB (NF-κB) is known to be a major signaling pathway activated by LPS in DCs (21), we hypothesized that NF-κB might play a role in LPS-stimulated CLEC-2 expression in DCs. Furthermore, computational analysis with the TFBIND program identified a putative NF-κB-binding site, ^−323^GGATACTCCC^−314^ (base positions are relative to ATG translation start site), in the mouse *CLEC1B* (which encodes CLEC-2) promoter (Fig. 4A). Therefore, we checked whether LPS induces NF-κB binding to the *CLEC1B* promoter in DCs. As evidenced by electrophoretic mobility shift assay (EMSA), LPS treatment increased the binding of nuclear protein (or proteins) to the mouse *CLEC1B* promoter-specific CLEC1B-Pr probe, which contained a potential NF-κB-binding site (Fig. 4A and B). However, mutation in the NF-κB-binding sequence prevented this nuclear protein binding (Fig. 4C). Moreover, in a DNA pulldown assay, the biotinylated CLEC1B-Pr oligonucleotide effectively precipitated the NF-κB subunits p65 and p50 from the nuclear preparations of LPS-treated BMDCs, whereas MutCLEC1B-Pr oligonucleotide did not (Fig. 4D). The latter oligonucleotide contained a mutated NF-κB site (Fig. 4A). Chromatin immunoprecipitation (ChIP) analyses further showed enhanced recruitment of p65 and p50 to the murine *CLEC1B* promoter upon LPS stimulation (Fig. 4E). Together, these results demonstrated the ability of LPS to induce NF-κB binding to the *CLEC1B* promoter in DCs.

Next, we performed a luciferase reporter assay to determine whether NF-κB regulates *CLEC1B* promoter activity. We observed that overexpression of NF-κB p65 in HEK-293T cells strongly increased wild-type *CLEC1B* promoter activity (Fig. S3). In contrast, overexpression of the IκBα dominant negative mutant (IκBαDN; NF-κB-specific inhibitor) markedly reduced this effect (Fig. S3). Similarly, NF-κB p65 overexpression failed to induce the *CLEC1B* promoter activity when we mutated the NF-κB-binding site in the *CLEC1B* promoter (Fig. S3). These data suggest that NF-κB plays a key role in driving *CLEC1B* promoter activity.

Further, to verify the role of NF-κB in LPS-stimulated CLEC-2 expression, we analyzed the effect of IκBαDN overexpression in BMDCs. While LPS-induced CLEC-2 upregulation was readily detected in control BMDCs (i.e., BMDCs left untransfected or transfected with the control vector), forced expression of IκBαDN in BMDCs blocked this effect (Fig. 4F). Indeed, overexpression of NF-κB alone was sufficient to promote CLEC-2 upregulation on DCs (Fig. 4G). These observations confirmed that NF-κB is required for LPS-mediated upregulation of CLEC-2 expression on DCs. Our results also showed that LDPm infection for 24 or 48 h largely inhibited LPS-stimulated NF-κB binding to the *CLEC1B* promoter in DCs and that this inhibition occurred to similar extents at both time points (Fig. 4H). Therefore, for subsequent experiments, we infected DCs with L. donovani for 24 h unless otherwise indicated. Next, we evaluated the role of TGF-β in L. donovani-induced suppression of NF-κB binding to the *CLEC1B* promoter. We found that the addition of neutralizing anti-TGF-β Ab considerably reduced the inhibitory effect of LDPm on LPS-induced binding of NF-κB to the *CLEC1B* promoter (Fig. 4I). Collectively, these results suggest that L. donovani suppresses CLEC-2 expression in DCs by preventing NF-κB binding to the *CLEC1B* promoter and that L. donovani mediates this inhibitory effect through TGF-β.

### TGF-β secretion induced by L. donovani reduces CLEC-2 expression on DCs by suppressing NF-κB through c-Src.

**FIG 5:**
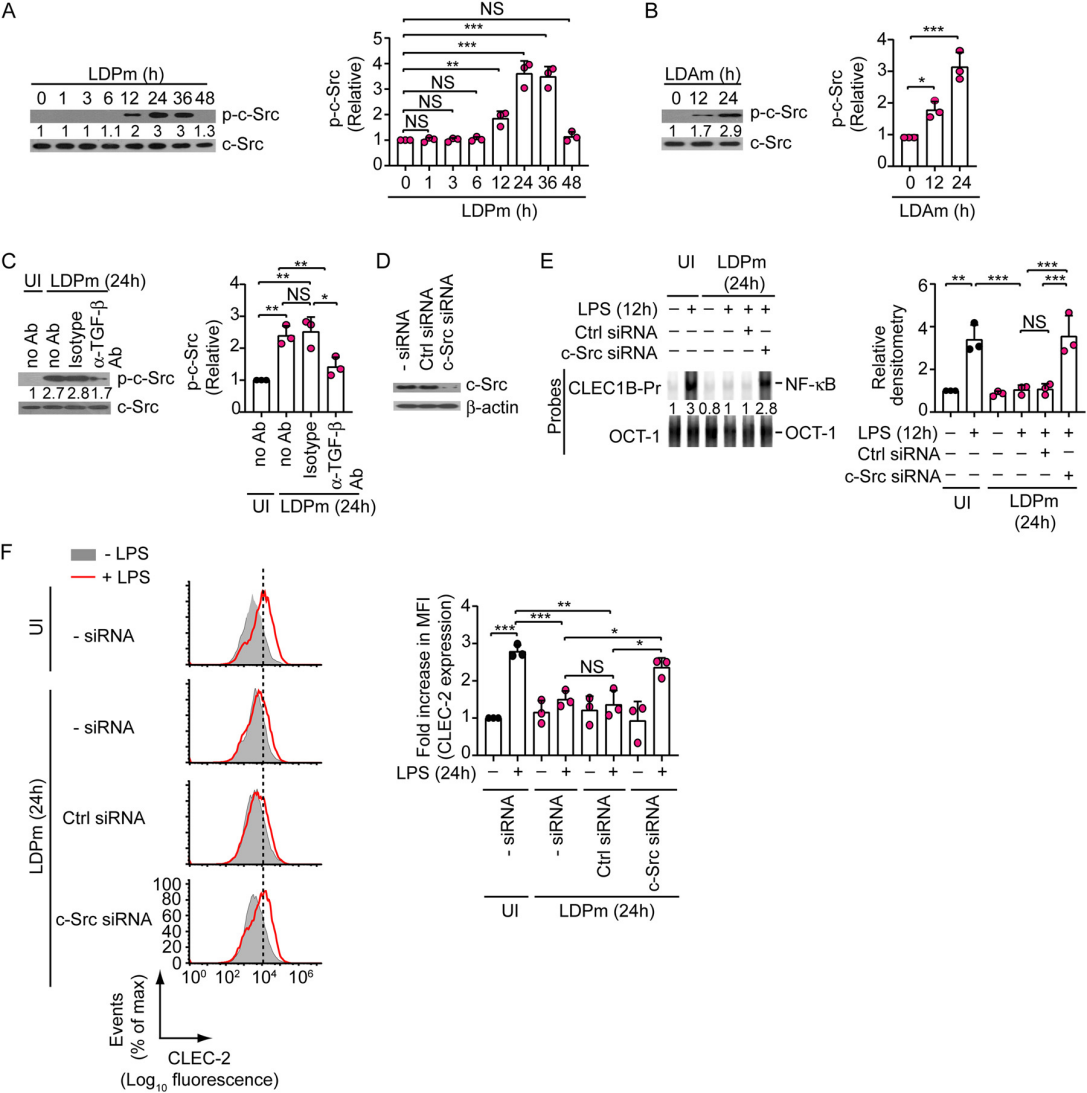
TGF-β induced by L. donovani suppresses CLEC-2 expression on DCs via c-Src. (A and B) Immunoblot analysis of the expression of total and phosphorylated (p-) c-Src in lysates of BMDCs infected with LDPm (A) or LDAm (B) for the indicated times. Numbers below lanes indicate densitometry normalized to total c-Src and presented relative to uninfected BMDCs (0 h). Data are representative of those from three experiments (left panel). (Right panel) Relative densitometry data compiled from three experiments. (C) BMDCs were infected with LDPm for 24 h in the presence of the indicated antibodies. The expression of total or phosphorylated c-Src was assessed via immunoblot analysis. Numbers below lanes indicate densitometry (as in panel A) presented relative to uninfected BMDCs cultured in the absence of Ab (no Ab). Data are representative of those from three individual experiments (left panel). (Right panel) Combined densitometry data (relative) from three different experiments. (D) Representative immunoblot (out of three separate experiments) showing the expression of c-Src and β-actin (loading control) in BMDCs left untransfected (-siRNA) or transfected with control siRNA or c-Src-specific siRNA. (E) BMDCs were transfected with siRNAs as for panel D and then infected with LDPm for 24 h or left uninfected and stimulated with LPS for 12 h. The binding of NF-κB to the *CLEC1B* promoter was determined via EMSA. Numbers below lanes indicate relative densitometry as in Fig. 4H. Data are representative of those from three independent experiments (left panel). (Right panel) Densitometry data pooled from three separate experiments. (F) Flow cytometry analysis of CLEC-2 expression on BMDCs transfected with the indicated siRNAs, then infected with LDPm for 24 h, and stimulated with LPS for another 24 h. Data are representative of three independent experiments (left panel). (Right panel) Relative mean fluorescence intensity of CLEC-2 expression (measured as for Fig. 1A) from three separate analyses. Error bars indicate SD. Each symbol in the graphs corresponds to data derived from an independent experiment. ***, *P < *0.001; **, *P < *0.01; *, *P < *0.05.

Previously, we have demonstrated that TGF-β suppresses CLEC2 expression on DCs via c-Src (5). Accordingly, we asked whether c-Src plays any role in TGF-β-mediated L. donovani-induced suppression of CLEC-2 expression in DCs. Initially, we determined whether L. donovani induces c-Src activation in DCs. To verify this aspect, we infected BMDCs with LDPm for various times and assessed c-Src activation by measuring c-Src Tyr 416 phosphorylation (5) by immunoblot analysis. We found that c-Src phosphorylation was considerably increased at 12 h to 36 h post-LDPm infection (Fig. 5A). Infection with LDAm similarly induced c-Src phosphorylation in DCs (Fig. 5B). These observations indicated the ability of L. donovani to activate c-Src in DCs. We then investigated whether L. donovani promoted c-Src activation in DCs in a TGF-β-dependent manner. To address this issue, we analyzed the effect of TGF-β neutralization (using anti-TGF-β Ab) on L. donovani-induced c-Src activation in DCs. Whereas LDPm infection efficiently triggered c-Src phosphorylation in BMDCs incubated with isotype control Ab, TGF-β neutralization with anti-TGF-β Ab suppressed this effect (Fig. 5C). Thus, L. donovani promoted c-Src activation in DCs in a TGF-β-dependent manner. We next evaluated the role of c-Src in L. donovani-induced inhibition of NF-κB binding to the *CLEC1B* promoter and downregulation of CLEC-2 expression in DCs. We observed that suppression of c-Src expression using small interfering RNA (siRNA) abrogated the inhibitory effect of L. donovani on LPS-stimulated NF-κB binding to the *CLEC1B* promoter and upregulation of CLEC-2 expression on DCs (Fig. 5D to F). Together, these results suggest that c-Src is necessary for L. donovani-induced inhibition of CLEC-2 expression in DCs.

### L. donovani-induced CLEC-2 downregulation via c-Src impedes DC migration to lymph nodes.

**FIG 6:**
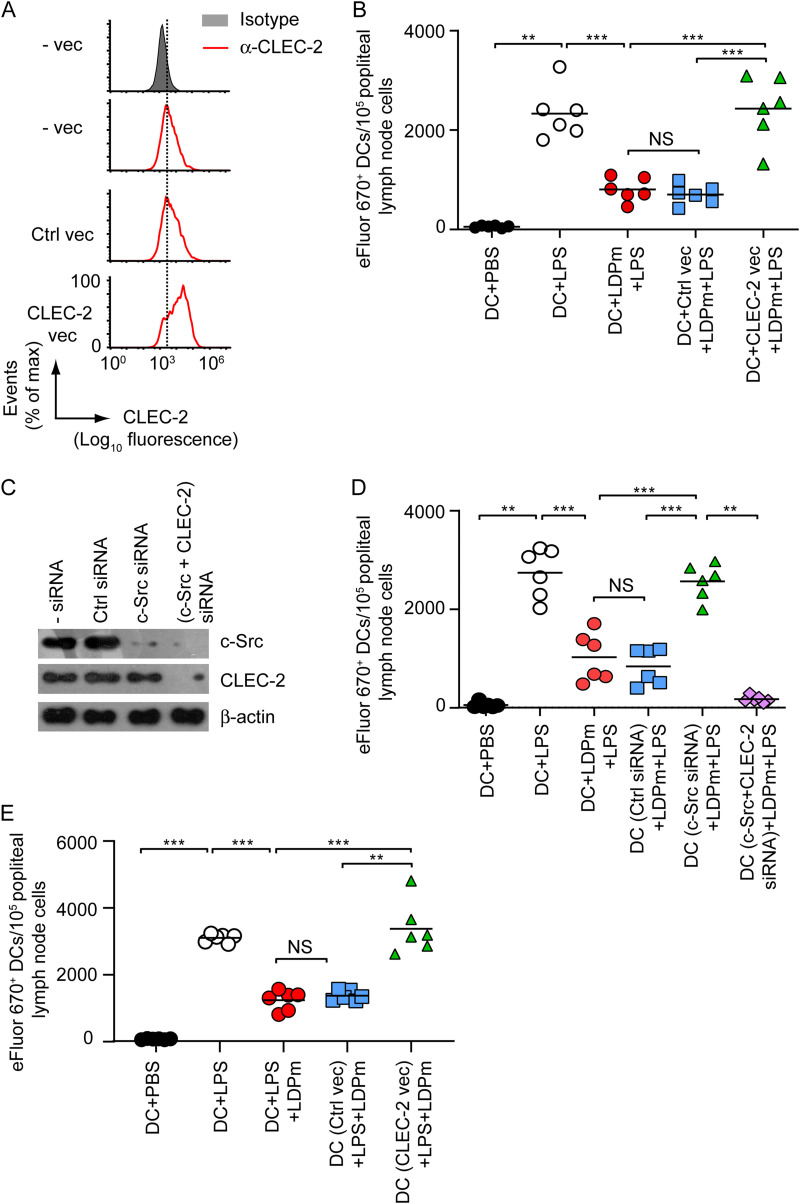
L. donovani inhibits migration of DCs to draining lymph nodes by reducing CLEC-2 expression via c-Src. (A) BALB/c BMDCs were transfected with a control vector (Ctrl vec) or CLEC-2-expressing vector (CLEC-2 vec) or left untransfected (-vec). The CLEC-2 expression on BMDCs was determined by flow cytometry. (B) BALB/c BMDCs (1 × 10^6^) were left uninfected or infected with LDPm for 24 h and then treated with LPS for another 24 h. Alternatively, BMDCs (1 × 10^6^) were transfected as for panel A before LDPm infection. In some sets, uninfected BMDCs were treated with PBS. BMDCs (1 × 10^6^) were then labeled with eFluor 670 dye and injected into the hind footpads of syngeneic mice. After 48 h, the number of injected DCs (i.e., eFluor 670^+^ cells) reaching the popliteal lymph nodes was determined via flow cytometry. (C) BALB/c BMDCs were kept untransfected (-siRNA) or transfected with control siRNA (Ctrl siRNA) or c-Src siRNA either alone or in combination with CLEC-2 siRNA. The expression of c-Src, CLEC-2, and β-actin was analyzed by immunoblotting. (D) BALB/c BMDCs (1 × 10^6^) were transfected with siRNAs as for panel C, infected with LDPm for 24 h, and then treated with LPS for an additional 24 h. DC migration to popliteal lymph nodes was assessed as described for panel B. (E) BALB/c BMDCs (1 × 10^6^) were transfected as for panel A and then treated (or not) with LPS for 24 h. Afterward, BMDCs (1 × 10^6^) were labeled with eFluor 670 dye, mixed with LDPm (1 × 10^7^), and immediately injected into the hind footpads of syngeneic mice. The migration of DCs to popliteal lymph nodes was evaluated as for panel B. Data in panels A and C are representative of those from two different experiments, and data presented in panels B, D, and E are a compilation of three separate experiments (*n *= 2 mice per group in each experiment). Each symbol in panels B, D, and E represents data of an individual mouse, and bars indicate means. ***, *P < *0.001; **, *P < *0.01; NS, not significant.

Having found that L. donovani inhibits DC migration by downregulating CLEC-2 expression (Fig. 1 and 3), we investigated whether L. donovani reduces the lymph node homing capacity of DCs and, if so, whether CLEC-2 and c-Src play any role in this process. First, we examined the effect of L. donovani infection on DC migration to lymph nodes. In addition, we determined the involvement of CLEC-2 in mediating such regulatory effect of L. donovani on DC migration. For these analyses, we left DCs uninfected or infected with LDPm, and treated them with LPS. In some sets, we overexpressed CLEC-2 in DCs (Fig. 6A) before LDPm infection. We then labeled these DCs with eFluor 670 dye, injected them subcutaneously into the hind footpads of syngeneic mice, and measured the number of injected DCs that migrated to the popliteal lymph nodes by flow cytometry. Compared to control DCs (phosphate-buffered saline [PBS]-treated DCs), LPS-treated DCs showed increased migration to popliteal lymph nodes (Fig. 6B). However, LDPm infection considerably attenuated this migratory ability of LPS-treated DCs (Fig. 6B). Overexpression of CLEC-2, on the other hand, restored the ability of LPS-treated DCs to migrate to popliteal lymph nodes despite LDPm infection (Fig. 6B). These findings, along with those shown in Fig. 1 and 3, suggest that L. donovani infection impairs the lymph node homing ability of DCs by reducing CLEC-2 expression.

Because L. donovani suppressed CLEC-2 expression through c-Src (Fig. 5F), we next investigated whether this c-Src-mediated CLEC-2 suppression contributed to L. donovani-induced inhibition of DC migration to lymph nodes. To investigate this issue, we silenced c-Src expression in DCs (Fig. 6C), then infected these DCs with LDPm, and stimulated them with LPS. Afterward, we evaluated DC migration to popliteal lymph nodes as described above. We found that silencing of c-Src expression markedly reduced LDPm’s inhibitory effect on LPS-treated DC migration (Fig. 6D). As a result, after c-Src silencing, LDPm-infected LPS-treated DCs migrated to popliteal lymph nodes with the same efficiency as LPS-treated DCs (Fig. 6D). Next, to see if c-Src silencing improved the migratory capacity of LDPm-infected LPS-treated DCs by increasing CLEC-2 levels, we cosilenced c-Src and CLEC-2 expression in these cells (Fig. 6C). We observed that cosilencing of c-Src and CLEC-2 completely blocked the migration of LDPm-infected LPS-treated DCs to popliteal lymph nodes (Fig. 6C and D). The latter finding ruled out the possibility that c-Src silencing augmented the migratory capacity of LDPm-infected LPS-treated DCs via a CLEC-2-independent mechanism. Together, these results suggest that L. donovani attenuates the lymph node homing ability of DCs by reducing CLEC-2 expression through c-Src.

Finally, we determined whether CLEC-2 downregulation was responsible for impaired DC migration to lymph nodes in L. donovani-infected mice. In this regard, it is noteworthy that mice infected with L. donovani via the standard intravenous route (20, 22–24) were not suitable for testing this hypothesis. This is because we studied DC trafficking from peripheral tissues to draining lymph nodes by injecting eFluor 670-labeled DCs into the hind footpads of syngeneic mice and analyzing the frequency of migrated DCs (eFluor 670^+^ DCs) in popliteal lymph nodes after 48 h. Unfortunately, we could not detect any parasite in the footpads of intravenously infected mice, although these parasites were readily detected in the spleen after 60 days of L. donovani infection (Fig. S4A). Notably, the presence of L. donovani parasites in the footpads was required to validate the aforementioned hypothesis, because inhibition of CLEC-2 expression and subsequent DC migration occurred only upon interaction with the parasites. Hence, we chose an alternative method of infecting mice with L. donovani by injecting the parasites into the hind footpads (25). Using this method, we were able to detect L. donovani parasites in the spleen within 1 day of infection (Fig. S4B). After confirming that L. donovani parasites injected into the hind footpads can eventually cause visceral infection, we followed this infection procedure to investigate the role of CLEC-2 in impaired DC migration to the lymph nodes in L. donovani-infected mice. Briefly, we treated BMDCs with PBS (control) or LPS and labeled them with eFluor 670 dye. In some experimental sets, we transfected BMDCs with a control vector or CLEC-2-expressing vector before LPS stimulation. We then mixed (or not) BMDCs with L. donovani parasites and immediately injected them into the hind footpads of syngeneic mice. This process allowed DCs to interact with L. donovani while residing within the footpads, just as they do with L. donovani deposited in the skin by sand flies. Notably, the DC-*Leishmania* coadministration process was also followed by other groups to assess DC migration to the draining lymph nodes (26). After 48 h of DC (with or without L. donovani) administration into hind footpads, we assessed the extent of DC migration to the popliteal lymph nodes by measuring the number of eFluor 670^+^ cells via flow cytometry. Our findings showed that LPS-treated BMDCs migrated to the popliteal lymph nodes at a higher rate than control BMDCs (Fig. 6E). However, the presence of L. donovani in the hind footpads markedly reduced the ability of LPS-treated BMDCs to reach popliteal lymph nodes (Fig. 6E). Importantly, overexpression of CLEC-2 efficiently promoted the migration of LPS-treated BMDCs despite the presence of L. donovani in the hind footpads (Fig. 6E). These results indicate that CLEC-2 downregulation affects the lymph node homing ability of DCs during L. donovani infection. Overall, our findings demonstrate the pivotal role of CLEC-2 and c-Src in L. donovani-induced impairment of DC trafficking to lymph nodes.

## DISCUSSION

A key pathological consequence of active VL is immunosuppression, which is partly mediated by defective DC migration (4). However, the molecular events that lead to impaired DC migration during L. donovani infection remain unclear. The current study has identified a new mechanism by which L. donovani attenuates the lymph node homing ability of DCs. Three key findings were derived from this study.

First, L. donovani reduces the migratory capacity of DCs to draining lymph nodes by downregulating CLEC-2 expression on DCs. CLEC-2 is a well-established mediator of DC trafficking (6). However, its role in L. donovani-induced inhibition of DC migration has remained unexplored. Additionally, it is still unknown whether L. donovani influences CLEC-2 expression on DCs. Our study has identified a new role of CLEC-2 that may be important in the context of host immunosuppression caused by L. donovani infection. In contrast to CLEC-2 downregulation on DCs, the frequency of PDPN-expressing LECs increased in mice during L. donovani infection. It may be possible that the increased PDPN expression by LECs in infected mice facilitates the migration of other CLEC-2-expressing cells, such as macrophages and neutrophils, that carry *Leishmania* parasites from the peripheral tissues to draining lymph nodes (27–31). Our findings further suggest that L. donovani reduces CLEC-2 expression on DCs by inducing TGF-β secretion from DCs. This conclusion can be drawn based on the observation that neutralization of TGF-β with anti-TGF-β Ab abrogated the inhibitory effect of L. donovani on CLEC-2 expression by DCs. A previous study has reported that L. donovani infection enhances TGF-β production by macrophages (32). Our data showed that TGF-β production was also induced in DCs in response to L. donovani infection. Moreover, by reducing CLEC-2 expression, TGF-β decreased the migratory ability of DCs. Indeed, we have shown here that overexpression of CLEC-2 restored the lymph node homing capacity of L. donovani-infected DCs. These findings demonstrate a crucial role for TGF-β and CLEC-2 in L. donovani-mediated impairment of DC migration. Unlike TGF-β, L. donovani-induced IL-10 production did not inhibit LPS-stimulated CLEC-2 upregulation on DCs. Although the latter finding indicates that the inhibition of CLEC-2 expression by L. donovani is TGF-β specific, we cannot completely exclude the possible involvement of other factors in mediating this inhibitory effect of L. donovani. Despite our above-mentioned observation that L. donovani-induced IL-10 production does not influence LPS-stimulated CLEC-2 expression on DCs, IL-10 nevertheless inhibits DC migration via a different mechanism. For example, IL-10 generated during L. donovani infection is reported to inhibit CCR7 expression on DCs and subsequent DC migration (4). A previous study has demonstrated that CLEC-2 mediates DC migration independent of CCR7 (6). Thus, our findings, together with the above-mentioned reports, depict two mutually nonexclusive mechanisms by which L. donovani impairs DC migration: (i) by downregulating CCR7 expression on DCs via IL-10 (4) and (ii) by reducing CLEC-2 expression on DCs via TGF-β (our current findings).

Second, our results provide evidence that L. donovani downregulates CLEC-2 expression on DCs by preventing NF-κB binding to the *CLEC1B* promoter. Although NF-κB is likely to participate in the CLEC-2-induced signaling pathway (33), its role in regulating CLEC-2 expression remains unknown. Our data showed that NF-κB overexpression alone promoted CLEC-2 upregulation on DCs. In addition, the blockade of NF-κB with IκBαDN greatly reduced LPS-stimulated CLEC-2 expression on DCs. These findings underscore the importance of NF-κB in promoting CLEC-2 upregulation on DCs. Notably, L. donovani infection mimicked the effect of IκBαDN in that LPS-stimulated CLEC-2 expression was also inhibited by L. donovani. Upon further investigation, we identified an NF-κB-binding site in the *CLEC1B* promoter. We have shown that LPS stimulation induced the binding of NF-κB to the *CLEC1B* promoter; however, L. donovani infection blocked this effect. These results define NF-κB as a key driver of CLEC-2 upregulation on DCs and demonstrate that L. donovani suppresses CLEC-2 expression in DCs by blocking NF-κB. Our data also showed that TGF-β was required for this L. donovani-induced inhibition of NF-κB binding to the *CLEC1B* promoter. Thus, TGF-β mediates the suppressive effect of L. donovani on CLEC-2 expression by DCs. Importantly, NF-κB activation in DCs is also inhibited by IL-10 produced during L. donovani infection (20). Based on this report from our group, one might question why L. donovani-induced IL-10 production failed to suppress CLEC-2 expression on DCs. In this context, it is noteworthy that IL-10 does not suppress all LPS-induced gene expression. Rather, IL-10 represses only 20 to 29% of LPS-stimulated gene expression (34–36). It should also be noted that IL-10, despite its inhibitory effect on NF-κB signaling, cannot always prevent LPS-induced NF-κB binding to target gene promoters. For example, IL-10 has been shown to prevent LPS-induced NF-κB binding to the *MHC-I* promoter in human monocytic cell lines THP-1 and U937 (37). However, in the same THP-1 and U937 cell lines, IL-10 fails to effectively suppress LPS-stimulated NF-κB binding to the *IL-6* promoter (38). These reports indicate that IL-10 inhibits LPS-induced NF-κB DNA binding activity in a gene-specific manner. As of now, it is unclear how IL-10 selectively suppresses LPS-induced NF-κB binding to certain gene promoters, and we are currently investigating this aspect. Nevertheless, the above reports explain why we did not detect the involvement of IL-10 in L. donovani-induced inhibition of LPS-stimulated CLEC-2 expression on DCs.

The third important finding from this study is that TGF-β produced during L. donovani infection suppresses CLEC-2 expression on DCs by inducing c-Src activation. When c-Src was suppressed, L. donovani failed to inhibit CLEC-2 expression on DCs. As a result, upon c-Src silencing, we found increased DC trafficking to lymph nodes despite L. donovani infection. These results are consistent with our previous report (5) demonstrating that TGF-β-mediated c-Src activation impedes DC migration by downregulating CLEC-2 expression. However, whether the same TGF-β/c-Src axis plays any role in L. donovani-mediated inhibition of DC migration has remained unaddressed so far. In light of this, our current findings document a previously unknown role for TGF-β/c-Src signaling in L. donovani-mediated impairment of DC migration. It should be noted here that the impairment of DC migration during L. donovani infection has also been reported by another group. Ato et al. have shown that migration of DCs to splenic lymphoid tissue is reduced in chronically L. donovani-infected mice (4). Similarly, DC migration to draining lymph nodes is significantly impaired in Leishmania major, Leishmania amazonensis, or Leishmania mexicana-infected mice (26, 39, 40). Indeed, *Leishmania* lipophosphoglycan (LPG) has been shown to reduce the migratory ability of Langerhans cells (LCs; a skin resident DC lineage) (41, 42). These reports support the notion that *Leishmania* parasites limit the lymph node homing potential of DCs, which is consistent with our findings.

But then, if DC migration from peripheral tissues to draining lymph nodes is impaired, how can *Leishmania* infection spread beyond the lymph nodes? This can be explained by the fact that DCs are not the only carriers of parasites or parasite antigens into the lymph nodes during *Leishmania* infection. Many other cells, such as macrophages and neutrophils, also phagocytose the parasites and then migrate to the draining lymph nodes (28, 30, 31, 43, 44). In fact, Baldwin et al. have reported that macrophages are the primary cells that transport *Leishmania* parasites from infected skin to draining lymph nodes, thereby promoting parasite dissemination (28). Unlike for macrophages, the latter study could not detect parasite-bearing DCs in the draining lymph nodes until 3 weeks after infection. It may be possible that skin resident DCs do not migrate out of the skin after infection because their migratory ability is impaired (28). Now, one might wonder how DCs can regulate the antileishmanial T cell response if these cells are unable to migrate to lymph nodes carrying parasites or parasite-derived antigens. This question is quite pertinent in view of the fact that DCs play a key role in initiating antileishmanial immune response. It is believed that DCs take up parasites released from infected macrophages in the lymph nodes but not parasites present in the skin (28). This may be one of the mechanisms by which DCs can regulate *Leishmania*-specific T cell reactivity. Alternatively, lymph node resident DCs, but not skin-derived DCs, acquire soluble *Leishmania* antigens through the lymph and present them to T cells (45). In this way, DCs can still play a role in regulating the antileishmanial T cell response. Nevertheless, impairing DC migration to lymph nodes may be a strategy used by parasites to subvert the development of adaptive immune responses.

**FIG 7:**
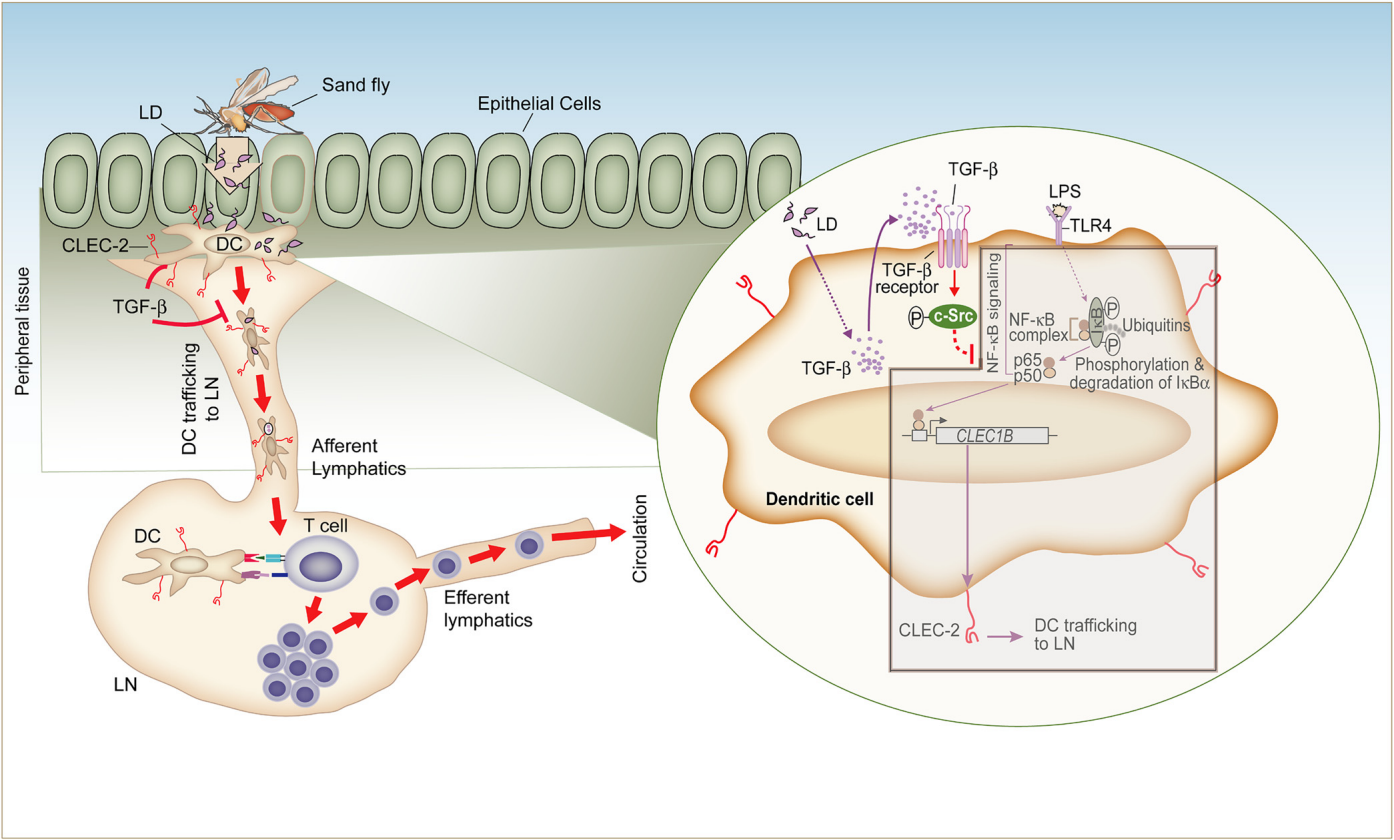
Model illustrating how TGF-β attenuates the lymph node homing capacity of DCs during L. donovani infection. Our results demonstrate a pivotal role of TGF-β and CLEC-2 (a lymph node homing receptor expressed by DCs) in L. donovani-induced impairment of DC migration to lymph nodes (LNs). During L. donovani infection, TGF-β is secreted by DCs. TGF-β then activates c-Src in DCs, which in turn prevents the binding of NF-κB to the *CLEC1B* (which encodes CLEC-2) promoter and thereby downregulates CLEC-2 expression on DCs (shown within circular inset). The reduced CLEC-2 expression significantly compromises the ability of DCs to migrate from peripheral tissue to draining lymph nodes and hence may attenuate antileishmanial T cell responses. Thus, by limiting the availability of DCs in lymph nodes, CLEC-2 downregulation mediated by the TGF-β/c-Src pathway contributes to immunosuppression during L. donovani infection. The brown-bordered box with the gray shaded area shows the events blocked by L. donovani-induced TGF-β secretion by DCs.

In conclusion, we have shown that L. donovani reduces the lymph node homing capacity of DCs by blocking NF-κB-mediated CLEC-2 expression on DCs. L. donovani exhibits such inhibitory effects by inducing c-Src activation through TGF-β. This inhibition of CLEC-2-mediated DC migration may, in turn, affect the adaptive T cell response (6) (Fig. 7). Overall, our findings have unraveled a new mechanism for L. donovani-mediated suppression of DC trafficking to lymph nodes and established a pivotal role for TGF-β, c-Src, and CLEC-2 in controlling this process. Notably, the role of TGF-β in VL is still understudied. It is generally believed that TGF-β plays a key role in inducing immunosuppression during VL (46). A suggested mechanism underlying this TGF-β-mediated immunosuppression is that TGF-β produced by antigen-presenting cells inhibits the *Leishmania* antigen-induced lymphoproliferative response (46). In addition, a recent report has proposed that TGF-β together with IL-35 inhibits antileishmanial T helper cell type 1 (Th1) responses (47). In this context, our study has revealed an “additional” mechanism by showing that TGF-β/c-Src signaling induced by L. donovani markedly decreases the arrival of DCs to lymph nodes by reducing CLEC-2 expression on DCs, which may subsequently limit the availability of DCs to prime T cells. Thus, targeting TGF-β (with anti-TGF-β Ab, e.g., metelimumab or GC1008, which are currently under phase I trial [48]) or its downstream effector c-Src (with clinically approved inhibitors [49]) may provide an approach for improving the antileishmanial adaptive immune response in VL patients. However, future research should evaluate the potential of these therapeutic options.

## MATERIALS AND METHODS

### Ethics statement.

The Biosafety Committee of the Institute of Microbial Technology approved the use of L. donovani for this study (IMTECH/IBSC/2018/19). All animal experiments were performed with the approval of the Institutional Animal Ethics Committee (IAEC/18/04 and IAEC/21/07) and in accordance with the National Regulatory Guidelines issued by the Committee for the Purpose of Control and Supervision of Experiments on Animals (CPSEA), Government of India.

### Reagents and plasmids.

The following antibodies were used for immunoblot analyses: anti-c-Src (2108; 1:1,000 dilution) and anti-phospho-Tyr416 c-Src (2101; 1:1,000 dilution; both from Cell Signaling Technology, Danvers, MA, USA), anti-CLEC-2 (ab90542; 1:1,000 dilution; Abcam, Cambridge, UK), and anti-NF-κB p65 (sc-8008; 1:1,000 dilution), anti-NF-κB p50 (sc-8414; 1:1,000 dilution), anti-β-actin (sc-47778; 1:1,000 dilution), and horseradish peroxidase-conjugated anti-rabbit (sc-2004; 1:2,000 dilution), anti-rat (sc-2032; 1:2,000 dilution), and anti-mouse (sc-2005; 1:2,000 dilution; all were from Santa Cruz Biotechnology, Dallas, TX, USA) antibodies. The same anti-NF-κB p65 and anti-NF-κB p50 antibodies and mouse IgG (sc-2025; from Santa Cruz Biotechnology) were used for ChIP assay. Phycoerythrin (PE)-labeled anti-mouse CLEC-2 (146103), allophycocyanin (APC)-labeled anti-mouse PDPN (127410), fluorescein isothiocyanate (FITC)-labeled anti-mouse CD11c (117306), FITC-labeled CD31 (160212), PE-labeled rat IgG2b,κ (400608), and APC-labeled mouse IgG2a,κ (400219), used for flow cytometry analysis, were obtained from BioLegend (San Diego, CA, USA). Neutralizing anti-IL-10 (505002; clone JES5-16E3) and rat IgG2b,κ (400643; isotype control Ab) were also obtained from BioLegend. APC-labeled anti-human CLEC-2 (FAB1718A) was purchased from R&D Systems (Minneapolis, MN, USA). Purified anti-human CLEC-2 (MABF957) was obtained from Merk KGa (Darmstadt, Germany). Neutralizing anti-TGF-β (16-9243-85; clone 1D11.16.8) and mouse IgG1,κ (14-4714-82; isotype control Ab) were purchased from Thermo Fisher Scientific (MA, USA). Recombinant mouse granulocyte-macrophage colony-stimulating factor (GM-CSF) and IL-4 were from Peprotech (Rehovot, Israel), recombinant mouse PDPN-Fc protein was from Biolegend, and recombinant human PDPN-Fc protein was from R&D Systems. The ON-TARGETplus nontargeting control pool siRNAs and SMARTpool siRNAs targeting mRNA encoding mouse c-Src and CLEC-2 were from Dharmacon (Lafayette, CO, USA). Escherichia coli LPS (serotype O111:B4) and other reagents were obtained from Sigma-Aldrich (India) unless indicated otherwise.

The pFLAG-CMV2 and pFLAG-NF-κB p65 plasmids were gifted by A. S. Baldwin (University of North Carolina at Chapel Hill, USA), the pGL3-Basic-luciferase and pGL3-(CAGA)_12_-luciferase reporter constructs were gifted by C. H. Heldin (Uppsala University, Sweden) (19), and the pCMV plasmid was gifted by Yuping Lai (East China Normal University, Shanghai, China) (50). pCMV-IκBαDN (631923) was purchased from TaKaRa Bio USA, Inc. (CA, USA), and the pCMV3 vector encoding murine CLEC-2 (MG50306-UT) was obtained from Sino Biological US Inc. (Houston, TX, USA).

### Animals and L. donovani parasites.

BALB/c mice and golden hamsters (Mesocricetus auratus) were maintained and bred under pathogen-free conditions at the animal house facility of the Institute of Microbial Technology. L. donovani strain AG83 (MHOM/IN/83/AG83) was maintained in male or female golden hamsters (4 to 6 weeks old) as described previously (51). Amastigotes were derived from infected hamster spleens, transformed into promastigotes, and cultured as described previously (52).

### DC preparation.

BMDCs were generated from male or female BALB/c mice (8 to 12 weeks old) as described previously (23). In some experiments, HuMoDCs, purchased from Lonza (CC-2701), were used.

### DC infection and treatment.

DCs (5 × 10^6^/well) were infected *in vitro* with promastigotes (stationary phase) or amastigotes of L. donovani at a parasite-to-DC ratio of 10:1 for the indicated times in RPMI 1640 complete medium (10% fetal bovine serum [FBS], l-glutamine, nonessential amino acids, sodium pyruvate, penicillin-streptomycin, and 2-mercaptoethanol) as described previously (23). Approximately 57% ± 1.8%, 64.51% ± 2%, 70.22% ± 1.6%, and 80.65% ± 4.2% of DCs were found to be infected after 6 h, 12 h, 24 h, and 48 h of incubation with LDPm, respectively (Fig. S1A). Furthermore, approximately 1,401.86 ± 189, 1,471.44 ± 99.55, 2,286.88 ± 254.9, and 3,920.08 ± 482 amastigotes were detected per 1,000 DCs at 6 h, 12 h, 24 h, and 48 h after incubation with LDPm, respectively (Fig. S1B). DCs were then stimulated with LPS (500 ng/mL) for specified times. Alternatively, DCs were kept uninfected and then stimulated with LPS. In some experiments, L. donovani infection was performed in the presence or absence of 10 μg/mL of neutralizing anti-TGF-β or anti-IL-10 Ab or respective isotype control Ab.

### DC transfection.

DCs were transfected with 2 μg of control plasmids or plasmids encoding NF-κB p65, IκBαDN, or CLEC-2 using FreeStyle MAX reagent (Thermo Fisher Scientific) following the manufacturer’s recommendation. Alternatively, DCs were transfected with various siRNAs (60 nM) using Lipofectamine RNAiMAX reagent (Thermo Fisher Scientific).

### EMSA and immunoblot analysis.

Nuclear extracts were made from DCs as illustrated previously (23, 53). EMSA was done as described previously (23, 53) using P^32^-labeled DNA probes mentioned in Table S1. An OCT-1 probe, 5′-TGTCGAATGCAAATCACTAGAA-3′, was used as a control (23). Immunoblot analysis was carried out as described previously (23). Densitometry analysis was done using Scion Image software (Scion Corporation, MD, USA).

### ChIP assay.

ChIP assay was done as described previously (53) using the ChIP-IT kit (Active Motif, CA, USA) and antibodies against the p50 or p65 subunit of NF-κB or mouse IgG. The immunoprecipitated DNA was then analyzed by quantitative real-time PCR (see Table S1 for primer details).

### DNA pulldown assay.

DNA pulldown assay of nuclear extracts of DCs was performed as described previously (54) using streptavidin (SA)-conjugated Dynabeads and specified biotinylated oligonucleotides (Table S1). The pulldown proteins were then subjected to immunoblot analysis.

### Reporter assay.

The pGL3-Basic reporter plasmid containing the wild-type or NF-κB site-mutated murine *CLEC1B* promoter fragment (region from −451 to −1; base positions are relative to the ATG translation start site) was synthesized and procured from Biomatik (ON, Canada). HEK-293T cells (1 × 10^6^) were transfected with a DNA mixture containing either of the above-mentioned reporter constructs (250 ng), the *Renilla* luciferase reporter plasmid (pRL-CMV, 250 ng; internal control), and pFlag-NF-κB p65 (250 ng) either alone or together with pCMV-IκBαDN (250 ng) using FreeStyle MAX reagent (Thermo Fisher Scientific). In some sets, the empty vectors pFlag-CMV2 and pCMV were used instead of pFlag-NF-κB p65 and pCMV-IκBαDN, respectively, as experimental controls. After 24 h, the luciferase activity in the cell lysates was assessed using a dual-luciferase reporter assay kit (Promega).

To determine the bioactivity of TGF-β secreted by L. donovani-infected DCs, a luciferase reporter-based assay system (16, 17) was used. Briefly, we cotransfected HEK-293T cells (0.5 × 10^6^ cells/well) with pRL-CMV (500 ng) and a pGL3-(CAGA)_12_-luciferase reporter (2 μg) (19) using Lipofectamine 2000 (Thermo Fisher Scientific). The latter plasmid contained 12 repeats of the TGF-β-responsive SMAD-binding element (CAGA). After 6 h, we incubated these cells with supernatants (50% of total medium) derived from uninfected BMDC or L. donovani-infected BMDC cultures (L. donovani infection was done for 24 h). The cells were cultured for 24 h. The luciferase activity of the cell lysates was then determined using a dual-luciferase reporter assay kit.

### Evaluation of CLEC-2 expression by sDCs and PDPN expression by LECs in L. donovani-infected mice.

BALB/c mice (4 to 6 weeks old) were intravenously infected with stationary-phase LDPm (1 × 10^7^ parasites/mouse) or left uninfected. Splenocytes were prepared from L. donovani-infected or uninfected mice at various days postinfection. The expression of CLEC-2 within the CD11c-gated population (i.e., sDCs) was analyzed by flow cytometry.

To determine PDPN expression by LECs, tissues adjacent to the popliteal lymph nodes and surrounding lymphatics were isolated from the hind limbs of L. donovani-infected mice on the indicated days after infection. Tissues were then subjected to collagenase/DNase digestion for 2 h at 37°C. Cells were then passed through a 45-μm cell strainer, washed, and resuspended in PBS. The expression of PDPN by LECs (CD31-gated cells [6]) was determined by flow cytometry.

### Transwell DC migration assay.

BMDCs were infected with LDPm for 24 h in the presence or absence of isotype control antibody or neutralizing anti-IL-10 or anti-TGF-β antibody or left uninfected. Alternatively, CLEC-2 was overexpressed in BMDCs before L. donovani infection. BMDCs were then treated with LPS for 24 h. In some sets, BMDCs were kept uninfected and untreated. BMDCs (1 × 10^6^ cells in 1 mL of medium) were then added to the upper Transwell insert (5-μm pore size; Corning Life Science, USA) of a 24-well Transwell plate and were allowed to migrate for 48 h to lower wells that were precoated (or not) with mouse PDPN-Fc protein (10 μg/mL, 200 μL) and contained RPMI 1640 complete medium. After 48 h, the number of migrated cells was counted by flow cytometry. The migration index was calculated as the number of DCs that had migrated to the PDPN-Fc-coated well divided by the number of untreated DCs (without LPS) that had migrated to the uncoated well (4, 55).

For some Transwell migration assays, HuMoDCs were used in which cells (0.5 × 10^6^) were infected with LDPm for 24 h in the presence of neutralizing anti-TGF-β Ab or isotype control Ab. HuMoDCs were then treated with LPS for 24 h. In some sets, uninfected HuMoDCs were treated with LPS for 24 h in the presence of 10 μg/mL of anti-human CLEC-2 Ab or isotype control Ab. Subsequently, HuMoDCs (0.5 × 10^6^ cells in 1 mL of medium) were added to the upper Transwell insert, and migration of HuMoDCs to the lower well coated with human PDPN-Fc protein was assessed as described above.

### *In vivo* DC migration assay.

DC migration assay was essentially performed as described previously (5). Briefly, BALB/c BMDCs (1 × 10^6^) were left uninfected or infected with LDPm for 24 h at a parasite-to-DC ratio of 10:1 and then treated with LPS for 24 h. In some experimental sets, BMDCs were transfected with a control or CLEC-2-expressing vector before LDPm infection. Alternatively, BMDCs were transfected with control siRNA, c-Src siRNA, or a mixture of c-Src and CLEC-2 siRNAs before LDPm infection. BMDCs were then washed thoroughly to remove free parasites. Afterward, BMDCs were labeled with eFluor 670 dye (10 μM; eBioscience, San Diego, CA, USA) and injected into the hind footpads of syngeneic mice. After 48 h, the arrival of DCs in the popliteal lymph nodes was quantified (by measuring the number of eFluor 670^+^ cells) via flow cytometry.

To assess DC migration in L. donovani-infected mice, BMDCs were treated with LPS for 24 h. In some cases, BMDCs were transfected with a control vector or CLEC-2-expressing vector before LPS treatment. BMDCs (1 × 10^6^) were then labeled with eFluor 670 dye, mixed with LDPm (1 × 10^7^), and immediately injected into the hind footpads of syngeneic mice. The number of DCs that arrived in the popliteal lymph nodes after 48 h was measured as mentioned above. Notably, inoculation via the hind footpads has been previously used by another group to infect mice with L. donovani (25). Following this procedure, parasite dissemination in the spleen was detected within 1 day after intrafootpad inoculation (Fig. S4B). In addition, the DC migration procedure described here was also used by others (26).

### Flow cytometry.

Flow cytometry was done with a C6 Accuri flow cytometer and FACSVerse (BD Biosciences). Data were analyzed with FlowJo software (Tree Star).

### Analysis of IL-10 and TGF-β production by DCs.

BMDCs (1 × 10^6^ cells/mL) were infected with LDPm or LDAm for various times as described above. The culture supernatants were assayed for IL-10 (88-7105-88; Thermo Fisher Scientific) and TGF-β (88-8350-88; Thermo Fisher Scientific) using enzyme-linked immunosorbent assay (ELISA) kits following the manufacturer’s instructions.

Alternatively, HuMoDCs (0.5 × 10^6^ cells in 500 mL of medium) were infected with LDPm for 24 h. The concentration of TGF-β in the supernatants from HuMoDC culture was then measured using TGF-β ELISA kit as described above.

### Statistical analysis.

One-way analysis of variance (ANOVA; SigmaPlot 11.0 program) was used for statistical analyses. A *P* value of <0.05 was considered significant.

### Data availability.

All relevant data are within the paper and supplemental material files.
