# Supplementary material for: Leishmania donovani Attenuates Dendritic Cell Trafficking to Lymph Nodes by Inhibiting C-Type Lectin Receptor 2 Expression via Transforming Growth Factor-β
Source: Microbiol Spectr. 2023 May 1;11(3):e04122-22. doi: 10.1128/spectrum.04122-22 (PMC10269552; doi:10.1128/spectrum.04122-22)
Supplement: Supplemental file 1 — Supplemental material. Download spectrum.04122-22-s0001.pdf, PDF file, 0.7 MB [file spectrum.04122-22-s0001.pdf]

SUPPLEMENTAL MATERIAL

SUPPLEMENTAL TABLE

Table S1 Details of primers and oligonucleotides used in this study

Primer sequences for ChIP-qPCR

Promoter	Regions amplified	Forward primer sequence (5'→3')	Reverse primer sequence (5'→3')
Mouse *CLEC1B*	-398 to -242	ggcatcagttcaattacgtg	gaaagagtagatgcgggg

All position numbers are relative to the translational (ATG) start sites (TSS).

Probes used for EMSA and DNA pull-down assays

Probes	Positions	Sequence (5'→3')
**Mouse *CLEC1B* promoter**		
CLEC1B-Pr Probe (containing wild-type NF-κB-binding site)	-332 to -302	CATTTGACAGGATACTCCCTCAGGACGGAAC
MutCLEC1B-Pr probe (containing mutated NF-κB-binding site)	-332 to -302	CATTTGACAGG*TCGAAG*CCTCAGGACGGAAC

All position numbers are relative to the translation (ATG) start sites; binding sites for the transcription factors are underlined and mutated bases are italicized.

SUPPLEMENTAL FIGURES

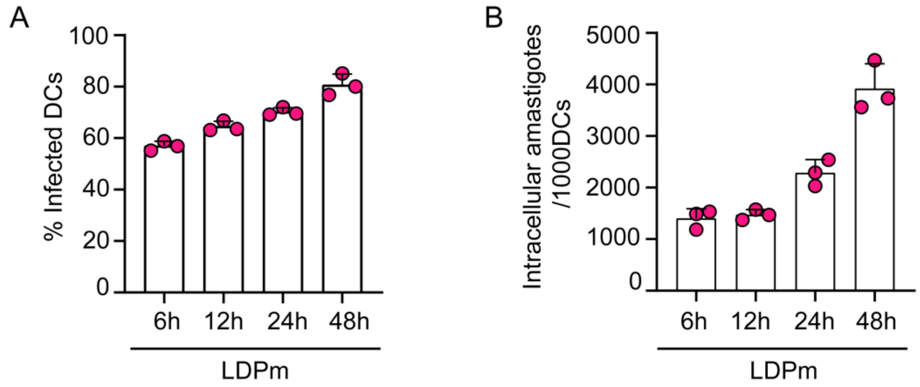

FIG S1 Analysis of *L. donovani* infection in DCs. BMDCs (1 x 10^6) were infected with LDPm (stationary phase) at a parasite-to-DC ratio of 10:1 for 6 h, 12 h, 24 h, and 48 h. The percentage of infected BMDCs (A) and the number of intracellular amastigotes per 1000 BMDCs (B) were determined by Giemsa staining and are presented here graphically. Data shown are a compilation of three independent experiments. In all panels, error bars indicate SD, and each symbol represents data derived from one independent experiment.

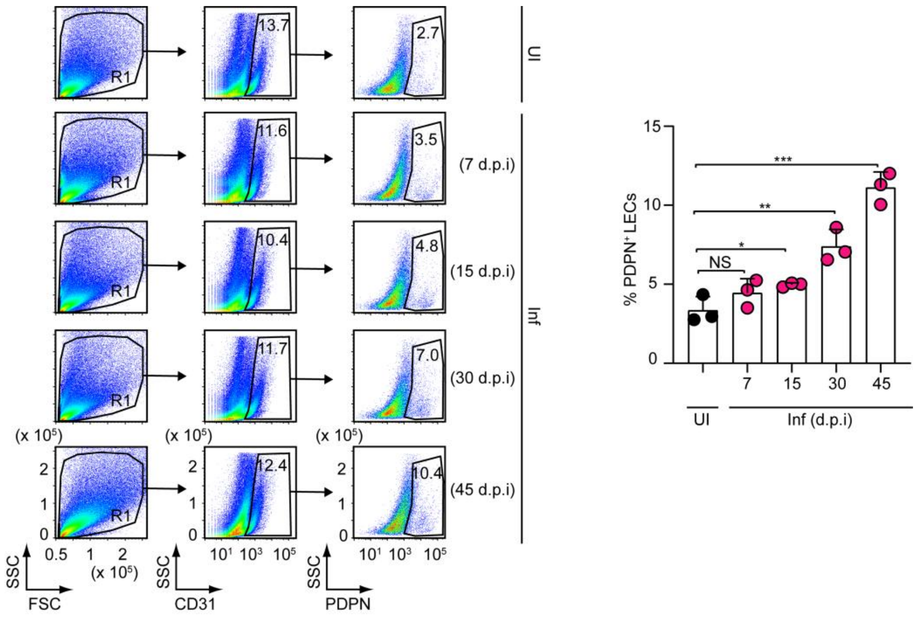

FIG S2 PDPN expression by LECs in *L. donovani*-infected mice. LECs derived from the hind limb of *L. donovani*-infected BALB/c mice on various days postinfection (d.p.i) were gated based on forward and side scatter, and CD31 expression. The frequency of PDPN-expressing LECs (i.e., CD31-gated cells) was analyzed via flow cytometry. Data presented in the left panel are representative of three experiments. The bar graph presented in the right panel shows compiled data derived from three separate experiments. UI, uninfected mice; Inf, infected mice. Error bars indicate SD. Each symbol in the graphs corresponds to data derived from an independent experiment. ***, $P < 0.001$; **, $P < 0.01$; *, $P < 0.05$; NS, not significant.

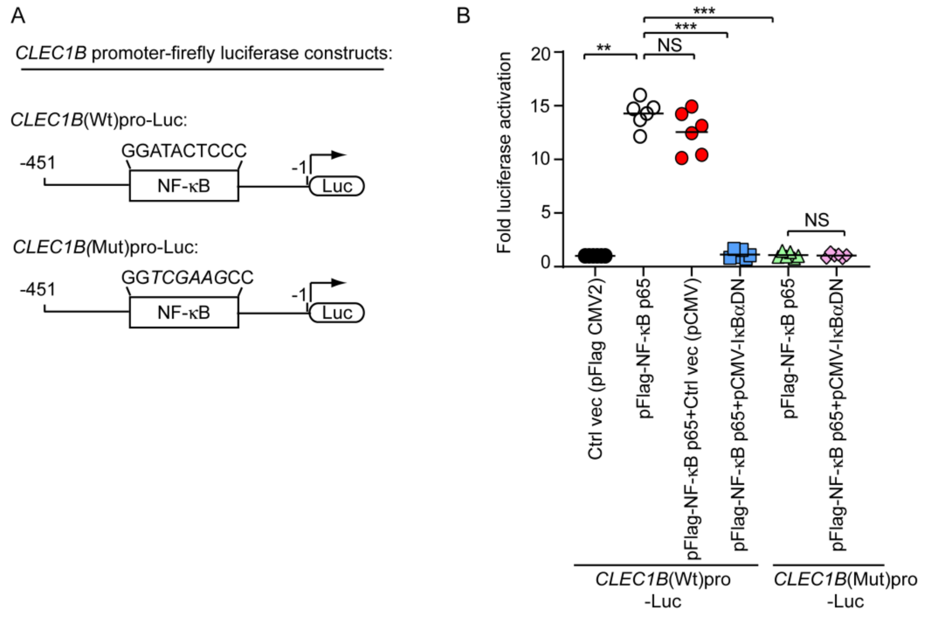

FIG S3 NF-κB regulates *CLEC1B* promoter activity. (A) Schematic presentation of the mouse *CLEC1B* promoter-firefly luciferase (Luc) reporter constructs used for promoter activity assay. The firefly luciferase reporter expression was driven by mouse *CLEC1B* promoter fragment (from -451 to -1 region; base positions are relative to ATG start site) that contained either wild-type or mutated NF-κB-binding site [represented as *CLEC1B*(Wt)pro and *CLEC1B*(Mut)pro, respectively]. (B) Luciferase activity of HEK-293T cells at 24 h after transfection with indicated luciferase reporter plasmids (as described in panel A) plus pRL-CMV (renilla luciferase plasmid; an internal control), and control vector (pFlag-CMV2) or NF-κB-encoding vector (pFlag-p65) either alone or in combination with a plasmid encoding IκBαDN (pCMV-IκBαDN) or respective empty vector (pCMV). Results were normalized to the activity of renilla luciferase and presented relative to that of cells transfected with wild-type *CLEC1B* reporter together with the control vector pFlag-CMV2. Compiled data from two experiments are presented here (n = 3 in each experiment). Horizontal bars represent means, and each symbol corresponds to data of individual replicate. ***, $P < 0.001$; **, $P < 0.01$; NS, not significant.

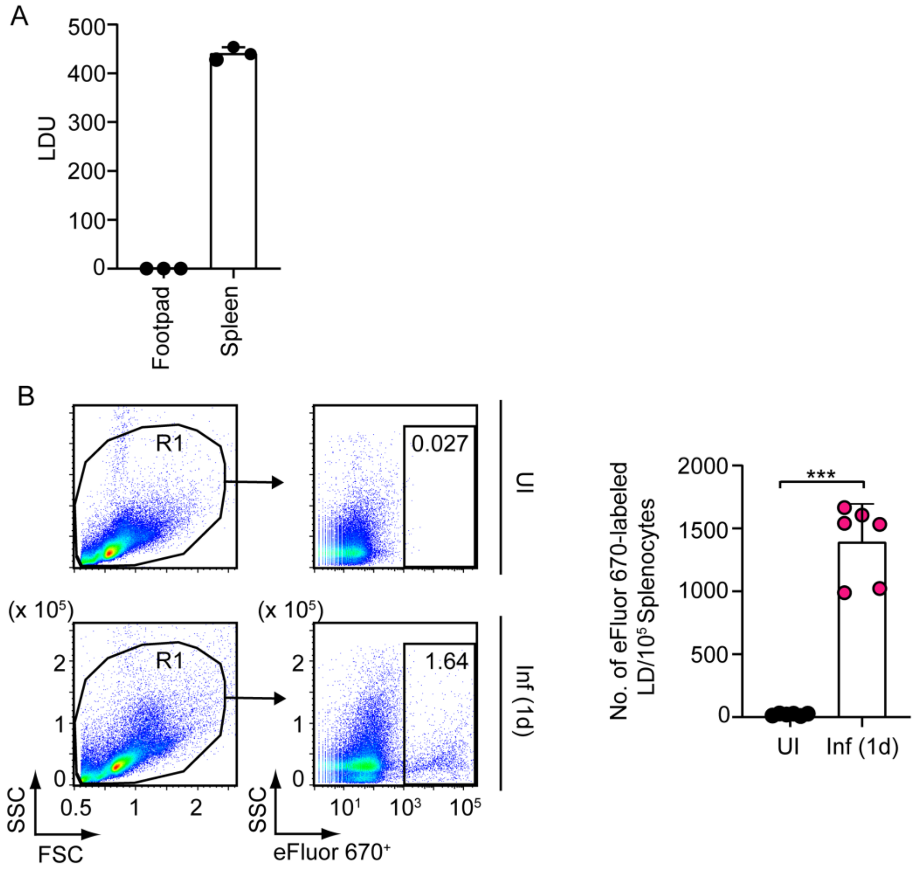

FIG S4 Detection of *L. donovani* parasites in the spleen and hind footpads after intravenous and footpad infection. (A) Spleen and hind footpad parasite load [expressed as Leishman-Donovan units (LDU); (1)] of BALB/c mice infected intravenously with LDPm (1 x 10^7); measured after 60 days. Data from n = 3 mice are presented. (B) LDPm (1 x 10^7) were labeled with eFluor 670 dye (10 μM) and injected into one of the hind footpads of BALB/c mice. After 1 day, the frequency (left panels) and number [per 1 x 10^5 splenocytes; right] of eFluor 670-labeled *L. donovani* parasites in the spleens were analyzed by flow cytometry (representative data of n = 6; left). UI, uninfected mice; Inf, mice infected through footpad. Numbers in the quadrants indicate percent eFluor 670-labeled parasites (left). Right, bar graphs show compiled data (n = 6 mice). Error bars indicate SD. Each symbol represents the data of an individual mouse. ***, $P < 0.001$.

REFERENCES

1. Saha B, Nanda-Roy H, Pakrashi A, Chakrabarti RN, Roy S. 1991. Immunobiological studies on experimental visceral leishmaniasis. I. Changes in lymphoid organs and their possible role in pathogenesis. Eur J Immunol 21:577-581. https://doi.org/10.1002/eji.1830210307.
